# Iron and manganese migration in “soil–plant” system in Scots pine stands in conditions of contamination by the steel plant’s emissions

**DOI:** 10.1038/s41598-020-68114-y

**Published:** 2020-07-03

**Authors:** Gleb A. Zaitsev, Olga A. Dubrovina, Ruslan I. Shainurov

**Affiliations:** 1 Laboratory of Forestry, Ufa Institute of Biology, Ufa Federal Research Centre of the Russian Academy of Sciences, pr. Oktyabrya 69, Ufa, Russia 450054; 2 Department of Chemistry and Biology, Institute of Mathematics, Science and Technology, Bunin Yelets State University, st. Kommunarov 28, Yelets, Russia 399770

**Keywords:** Forest ecology, Forestry, Environmental impact

## Abstract

In this paper, Scots pine (*Pinus sylvestris* L.) roots grown in soils with and without contamination from emission of a plant steel were analyzed for Fe and Mn, as well as the shoots and needles with and lacking pollution. The aim was to assess the content of Fe and Mn in soils under given conditions, and the interaction between pine plant and soil in terms of metal accumulation in the fine roots, annual shoots, and annual needles. The iron content in the soil of polluted areas does not contrast with its control amount. Conversely, the iron content in fine pine roots under contamination conditions is 2.1–4.4 times higher than the control values. There were no significant excesses of the manganese content in the soil in polluted conditions compared to the control, but its content in the 0–20 cm soil layer is 27–32 times higher than the background concentrations. The iron contentment in belowground (fine roots) and aboveground (annual shoots and needles) parts of pine trees in a context of contamination is higher than the control values (2.1–4.4 and 1.50–1.54 times, respectively). The manganese content in fine pine roots under contamination conditions is 2.8–10.7 times less than in control, while its content in shoots and needles is higher (2.23–2.76 times) in comparison with the control. Based on the values of the biological accumulation and migration coefficients, what in each case slighter than one, for Scots pine the iron represent not an element that actively accumulates. Nevertheless, for manganese, this stock model is valid only for fine roots, whereas under the contaminated environment, the metal mobility steepen, and the migration pattern shifts towards increased manganese accumulation in the aboveground part of pine trees.

## Introduction

The areas around the metallurgical plants consistently affected by airborne emissions containing various waste matters including toxic heavy metals. Toxicants cause direct damages to the aboveground part of trees, accelerates decline and eventual death^1^. The heavy metal accumulations in the soil represent, besides, a critical, damaging reason that affected in the growth process of trees. Within the soil profile, they exert influence on trees through direct toxic effects on the roots and indirect, altering the habitat of the roots. Toxicants, first, have a negative effect on the fine roots of trees^2–5^. Fine roots represent a key role in the forest ecosystem since they are responsible for a nutrient and water acquisition of trees. However, there is a very little known about their role in tree adaptation to high-level pollution conditions. A numeral of investigations has revealed (e.g.,^6–13^) that beneath conditions of pollution, the distribution of fine tree root mass along the soil profile changes and the portion of dead roots increases.

Iron and manganese are present in the emissions (as a particulate matter) at all stages of the metallurgical manufacturing cycle, from ore processing (iron and manganese ores treatment) to cast iron and steel production^14,15^. Aside from that, iron represents the commonest element on Earth and the most frequent utilized transition metal in the biosphere^16^. Manganese are detected naturally in many types of soil and other components of the environment (consistently presents in low levels in water and air)^17^.

Iron is essential for the production of hemoglobin, myoglobin, several essential enzymes, and involved in DNA synthesis. Iron is unique among metals because the human body possesses no mechanism for iron excretion, and excessive iron accruement can therefore result in iron poisoning^18^. When iron exceeds the required amount, it is stored in the liver. Chronic inhalation of excessive concentrations of iron oxide dusts may result in development of a benign pneumoconiosis^19^. High dossage of iron may cause conjunctivitis and choroiditis^20^.

Manganese nets a vital trace element necessary for various biological processes, but its lack, as well as excess has a negative impact on human health. The brain is the main target organ for the accumulation of manganese and the ostent of its toxicity. Action of excessive concentrations of manganese can lead to manganese poisoning (manganism) and Parkinson's disease^21,22^. May cause damage to lungs with repeated or prolonged exposure. Therefore, controlling the ingress and migration of manganese in the environment become a notable public health issue, especially for people living in regions with increased emissions of this metal^23^.

Aspects of the heavy metal contents in the soils of the Lipetsk region were fragmentarily examined^24,25^, but there is not very much information on iron and manganese migration along soil profile and specific features of their accumulation by the pine trees. The purpose of the present study is to contribute with quantitative information to the general knowledge of Scots pine (*Pinus sylvestris* L.) root systems in terms of the accumulation of some metals, and data of metal migrations from fine roots to annual shoots and needles.

## Materials and methods

### Site description

The investigation areas were located in Lipetsk city, Lipetsk Region, Russia. Region located in the junction zone of the Central Russian Upland and the Oka-Don Lowland^26^. Region climate in common is a moderate continental. The mean January temperature is − 8 °C and the mean July temperature is + 20 °C; mean annual precipitation is about 500 mm with a maximum in July. Northwest (warm season) and southwest winds (cold period) prevail. Located on the terraces above the flood-plain of Voronezh River, these sites are distinguished by light-gray forest soil on alluvial (sandy-loam and light-loamy) deposits with light texture. The Lipetsk region is identified by the low forest cover (the total forest area exist only of 7.6% of the territory), the part of natural forests is 53.8% (other 46.2% are the man-made forest stands). The share of pine stands (natural and man-made) explain 34% of the forested area.

Lipetsk represents the vastest city within the region (510 thousand inhabitants). The city possesses one of the largest Russians steel mills, the Novolipetsk Steel, which accounts for 86.2% of all atmospheric emissions from industrial enterprises in the region. Emissions into the atmosphere of pollutants and other substances from the Novolipetsk Steel in 2018 amounted to 275.97 thousand tons^27^. Into the structure of smelter's emissions, the following substances are predominating: carbon dioxide, particulate matter (dust), and nitrogen oxides.

### Data collection

**Figure 1:**
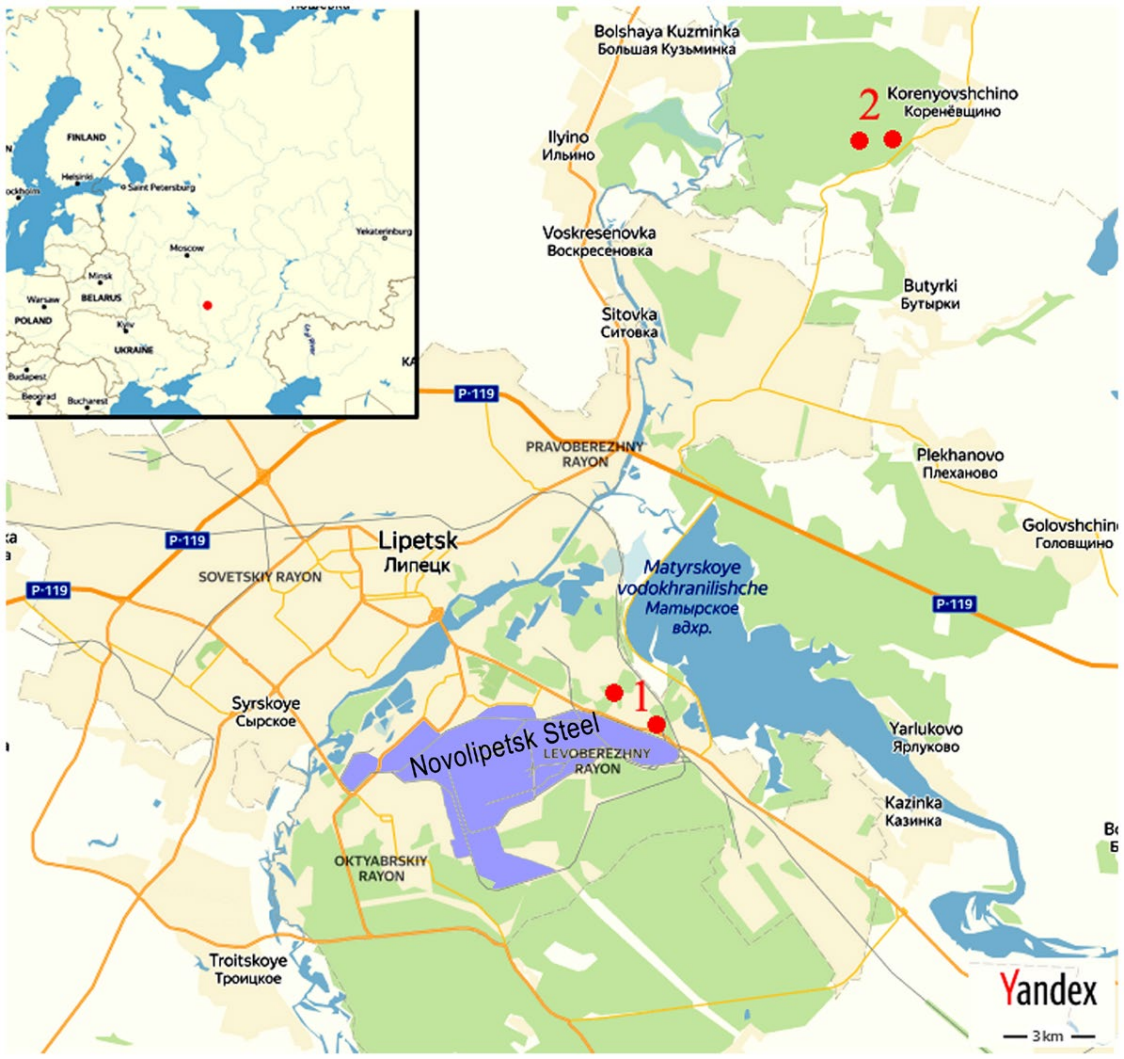
Location of the study area (1—impact zone, 2—control).

Experimental plots were laid in Scots pine cultures in sanitary-protective plantations and located in the close quarters of the Novlipetsk Steel (impact zone), near the ore-processing factory (the distance to the source of contamination was 300–400 m). As a control, we were able to locate two suitable pine stands (Fig. 1). These stands are in man-made forests located 17 km northeast of Lipetsk. Stands selected were of the comparable age (38–45 years old) and similar structure. Since these stands are artificially generated and located in the same forest compartment in each observed environment, they were created simultaneously, had similar planting material, and the identical forest engineering procedures, such as clean cutting, were carried out throughout their lives. Therefore, within the same zone (impact zone or control) between experimental plots, there are no significant differences in taxation characteristics. The average diameter at breast height (dbh) of the trees was 30.5 ± 0.7 cm (mean ± standard error, henceforward) and 31.4 ± 0.5 cm (polluted areas and control, respectively), average tree height was 29.1 ± 0.2 m and 31.2 ± 0.4 m. All sampling was done in two 10 × 10 m plots (a plot included not less than three trees) in each stand (both in polluted areas and in control) with a total number of four plots. Soil and root samples were collected by an auger method^28,29^ with 3.5 cm core diameter at five depths: 0–10, 10–20, 20–30, 30–40, and 40–50 cm. Ten cores were randomly picked up from each plot (i.e., total 40 soil cores). Shoots and needles were taken randomly from the basal part of the crown without the irrespective to the horizon direction or the pollution-exposed side^30^. Only needles and shoots of the current year’s growth (annual) were selected for research. At the minimum 25 shoots and 100 needles have been sampled from each experimental plot (i.e., total 100 shoots and 400 needles). Sampling campaigns were carried out in August–September 2019 (desition of the growing season).

### Chemical analysis

Soil pH (KCl) was determined using fresh soil samples. About 30 g of soil was placed in a 200-ml beaker, a 75 ml of 1 N KCl solution was added, this solution were then stirred in a rotary mixer for 3 min for organic soil layers and 1 min for other layers. The acidity was measured with a pH meter (Hanna pH 211)^31,32^. The humus amount estimated using Tyurin’s method^33^, when the soil organic matter was wet combustion by potassium dichromate (K_2_Cr_2_O_7_) solution in sulfuric acid (H_2_SO_4_) followed by determination of oxidizer value by titering with Mohr’s salt (ammonium iron(II) sulfate, (NH_4_)_2_Fe(SO_4_)_2_(H_2_O)_6_).

Before metal content estimation soil and non-organic material was careful washed away from roots by running tap water and were then manually separated from organic debris. To purge off the root surface from exchangeable manganese as well as iron plaque, pine roots were immersed into 0.2% ethylenediaminetetraacetic acid (EDTA, (HOOCCH_2_)_2_N(CH_2_)_2_N(CH_2_COOH)_2_ (C_10_H_16_N_2_O_8_)) for 2 h, then rinsed with deionized water^34–37^. Roots were carefully sorted into the diameter classes of fine (< 1 mm) and coarse roots (> 1 mm). Only fine roots (< 1 mm) were collected and included in this study.

Since the needle (leaf) surface can absorb water during rinsing^38^, the aboveground materials were rapid washed away from dust with 250 mL distilled aqua for 60 s^39–41^, after that samples were immediately placed on absorbing paper then transfer to the dry-off oven.

All soil and plant samples oven dried at 80 °C to a constant mass and were mineralized by dry ashing. The mobile forms of iron and manganese were extracted with 1 M HNO_3_. The metal content was determined by atomic absorption spectrometry^42^ using an atomic absorption spectrometer (Spektr-5). Since manganese is one of the metals that are controlled by environmental organizations and for which admissible concentration limits are calculated, its background concentrations were taken from pursuant to published data and regulatory documents^43^.

To describe the stock of elements in pine fine roots, we used the biological accumulation coefficient (BAC), which was calculated as a ratio of metal content in plant organs to that in soil^44–46^. The higher the coefficient value, the more intense the plant accumulates this metal. The BAC indicates the ability of plants to tolerate and accumulate heavy metals^47,48^. The criteria to define plants as hyperaccumulators the BAC mast be greater than one^49^. Other authors (e.g.,^50–54^) also point out that the plants actively accumulate metals if the BAC values are greater than one. To define the movement of metals within the pine plants, we applied the biological migration coefficient (BMC), which was counted as a ratio of element content in plant organs to that in roots^55^. The metal content value in the roots for calculating the BMC was taken as a mean from all soil layers. The more intense the metal movement in the plant, the higher the BMC value.

### Statistical analysis

All data getting from experiments was undergoing statistical processing. All analyses were performed using MS Excel and Graph Pad statistical software^56^, applied mathematics tests were achieved at a 0.05 significance level. The metal accumulation patterns in “soil-fine root” system were established through using regression models, and the selection criteria for the best models were a maximum adjusted *R*^2^ value. All figures were produced by Excel 2007 software. The map (Fig. 1) was generated by GIMP 2.10.10 GNU Image Manipulation Program (The free & open source image editor, https://www.gimp.org/), the map source—Yandex.Maps Service (open access license: Terms of use for Yandex.Maps Service, https://yandex.ru/legal/maps_termsofuse/?lang=en).

## Results

**Figure 2:**
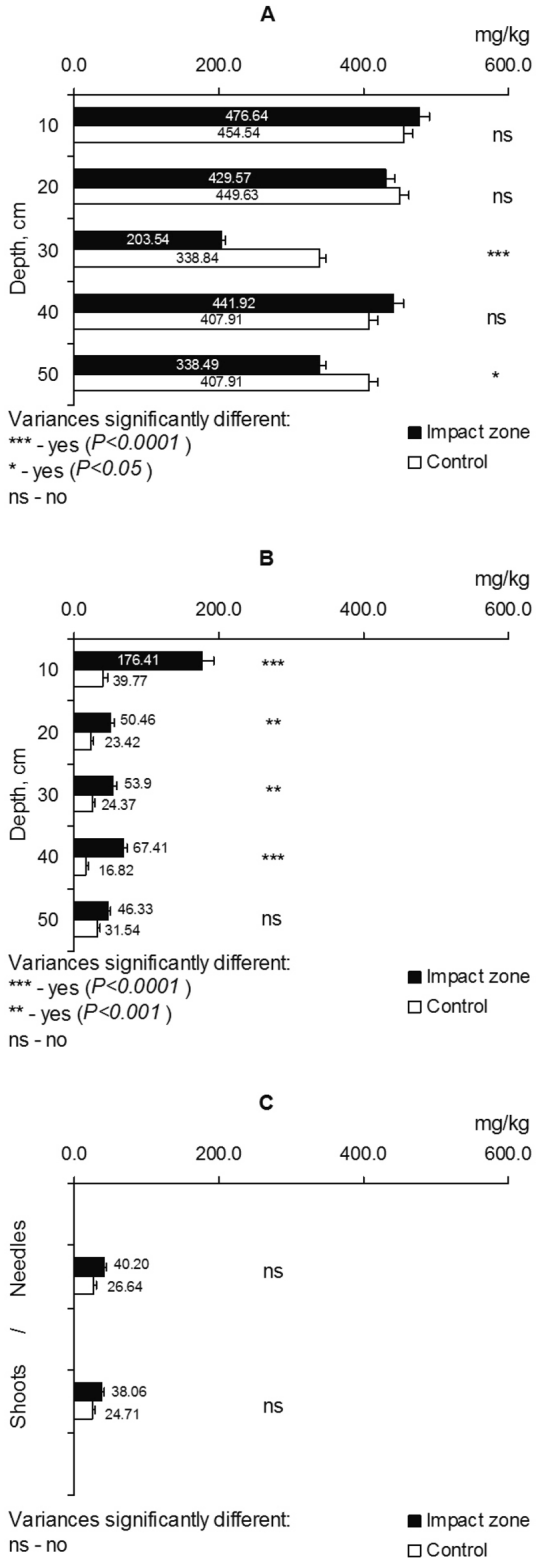
The iron content in the soil (**A**), fine roots (**B**), shoots and needles (**C**) of Scots pine (*Pinus sylvestris* L.).

The soils of the sampling plots in the contaminated areas are specified by an allied to neutral acidity and a huger humus content compared to the control (Table 1). The iron content in the soil layers 0–20 and 30–40 cm in polluted areas (impact zone) does not contrast with the level of its amount in control, but for other layers, the iron contents differ significantly (impact zone vs control) (Fig. 2A). Conversely, the iron content in fine pine roots (Fig. 2B) under contamination conditions is 2.1–4.4 times higher than the control values. The iron content in the shoots and needles in polluted areas is moderately higher (1.50–1.54 times) than in control, but these differences (impact zone vs control) are not statistically significant (Fig. 2C).

**Table 1:** The acidity level and humus content in the soils of the sampling plots.

Depth, cm	pH (KCl)		Humus, %	
	Impact zone	Control	Impact zone	Control
0–10	7.13	4.42	3.7	2.3
10–20	7.09	4.23	3.2	1.5
20–30	7.02	4.18	1.2	0.9
30–40	7.01	4.15	1.4	0.9
40–50	6.70	4.09	1.3	0.9

**Figure 3:**
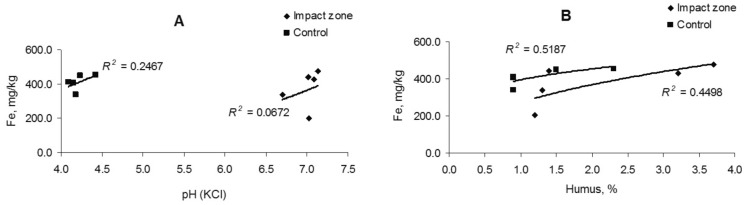
Dependence of iron content in soil on acidity (**A**) and humus amount (**B**).

**Figure 4:**
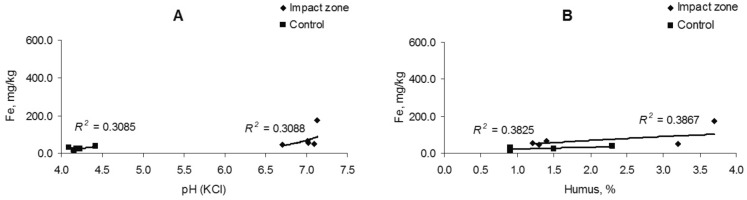
Dependence of iron content in fine roots on acidity (**A**) and humus amount (**B**).

The change in the iron content in the soil is uncorrelated (*R*^2^ = 0.06–0.24) with soil acidity, but there is a weak relationship (*R*^2^ = 0.44–0.51) with the amount of humus (Fig. 3). The iron content in fine roots is also weak correlated with soil acidity (*R*^2^ = 0.30) and humus content (*R*^2^ = 0.38) (Fig. 4).

**Figure 5:**
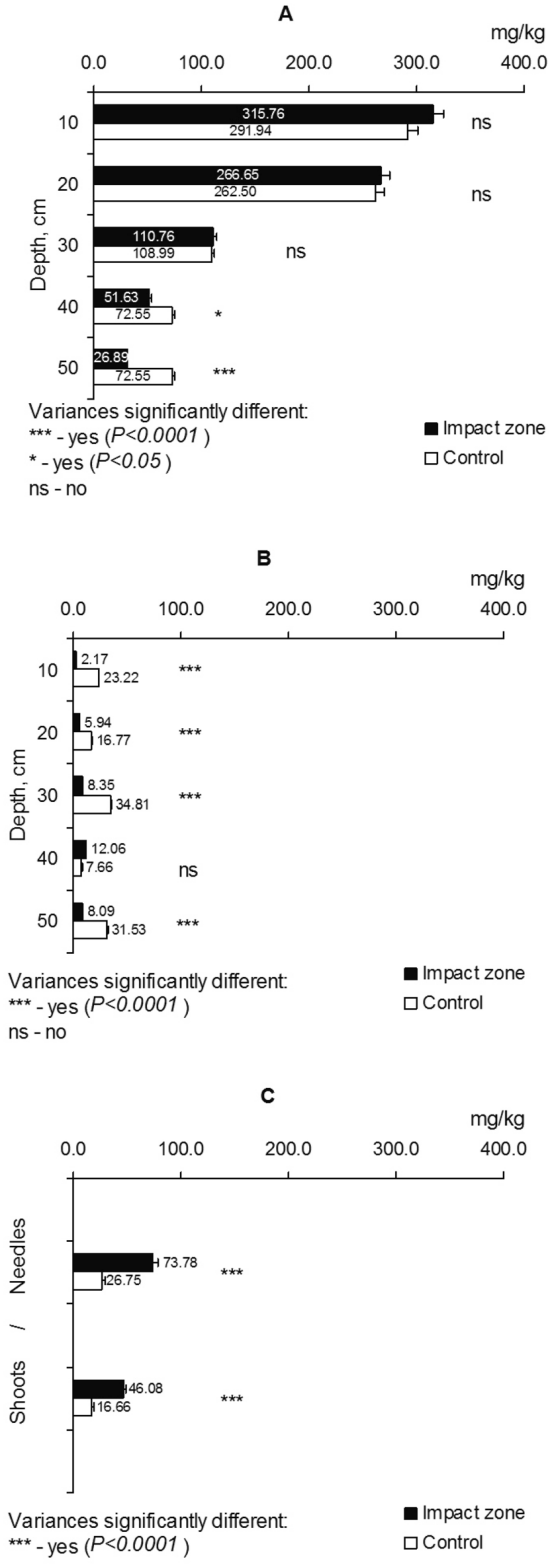
The manganese content in the soil (**A**), fine roots (**B**), shoots and needles (**C**) of Scots pine (*Pinus sylvestris* L.).

For the soil layer 0–30 cm, comparing absolute values (mg/kg), there were no significant excesses of the manganese content in the soil in polluted conditions compared to the control, but for the layer 30–40 and 40–50 cm, the Mn content differs significantly (Fig. 5A). Nevertheless, when comparing the manganese content accompanied by the background values, then its content in the 0–20 cm soil layer is 27–32 times higher than the background concentrations. The manganese content in fine pine roots under contamination conditions is 2.8–10.7 times less than in control (except for depths of 30–40 cm) (Fig. 5B). The manganese content in the shoots as well as needles in polluted plots is higher (2.23–2.76 times) in comparison with the control, and these differences (impact zone vs control) are statistically significant (Fig. 5C).

**Figure 6:**
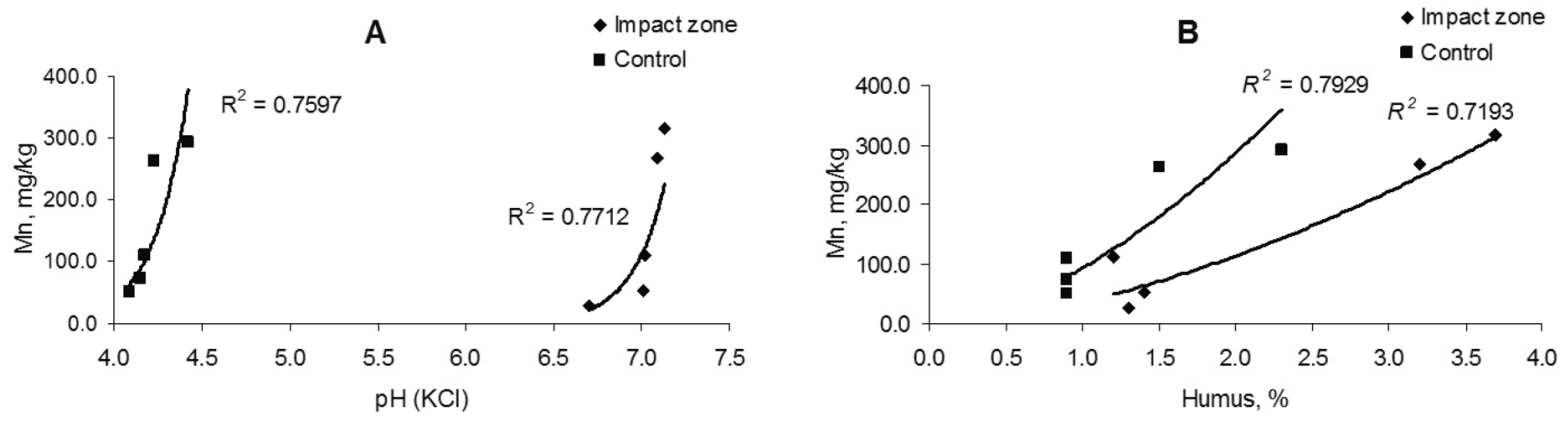
Dependence of manganese content in soil on acidity (**A**) and humus amount (**B**).

**Figure 7:**
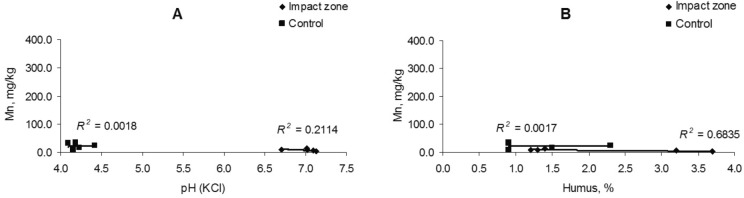
Dependence of manganese content in fine roots on acidity (**A**) and humus amount (**B**).

There take place a significant correlation (a strong uphill (positive) relationship) of the manganese content in the soil with the acidity (*R*^2^ = 0.75–0.77) and amount of humus (*R*^2^ = 0.71–0.79) (Fig. 6). The manganese content in fine roots is uncorrelated with soil acidity (*R*^2^ = 0.001–0.211) and the amount of humus (*R*^2^ = 0.001), except for the ratio of its content in polluted conditions to the humus quantity that is characterized by a moderate correlation (*R*^2^ = 0.68) (Fig. 7).

**Figure 8:**
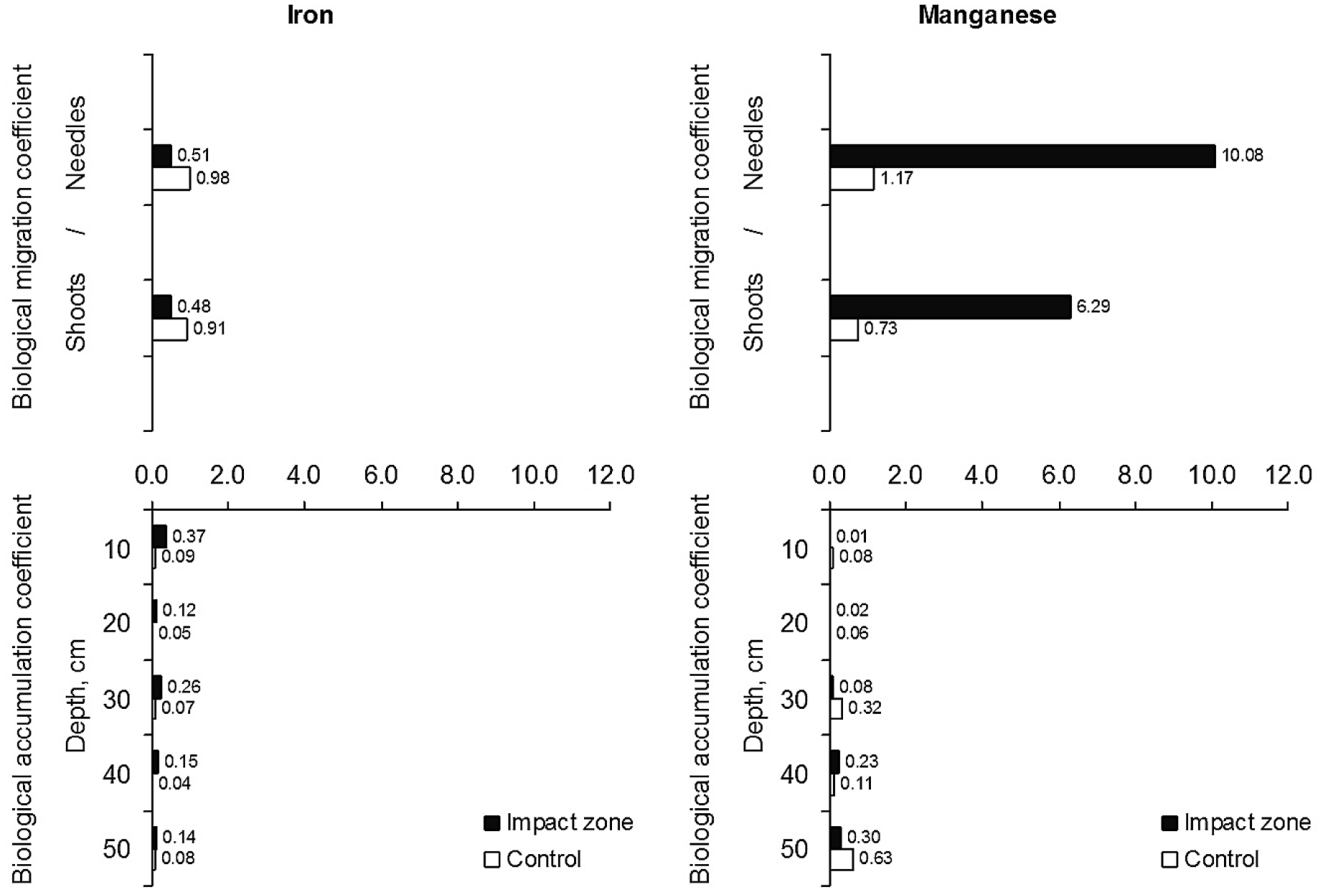
The biological accumulation and biological migration coefficients of iron and manganese.

The BAC of iron in contaminated conditions differs from 0.12 to 0.37, in control – from 0.04 to 0.09; the BMC value for needles under contamination is 0.51 and in control – 0.98, for shoots – 0.48 and 0.91, respectively (Fig. 8A). The BAC of manganese in polluted areas varies from 0.01 to 0.30, in control – from 0.06 to 0.63; the BMC value for needles in a context of contamination is 10.08 and in control – 1.17, for shoots – 6.29 and 0.73, respectively (Fig. 8B).

**Figure 9:**
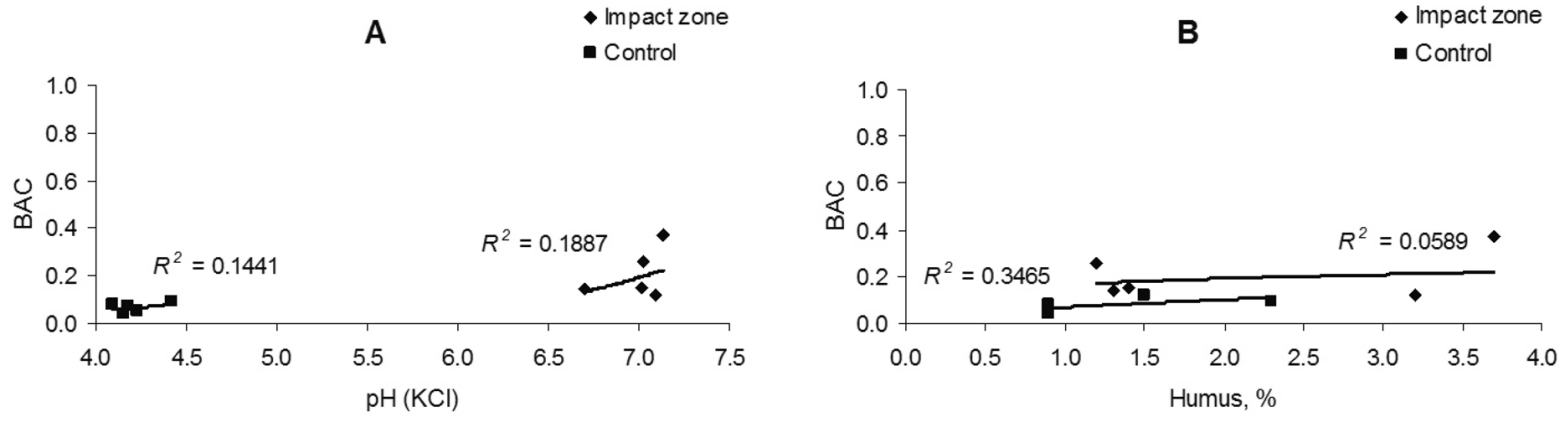
Dependence of the biological accumulation coefficient of iron on the acidity (**A**) and humus amount (**B**).

**Figure 10:**
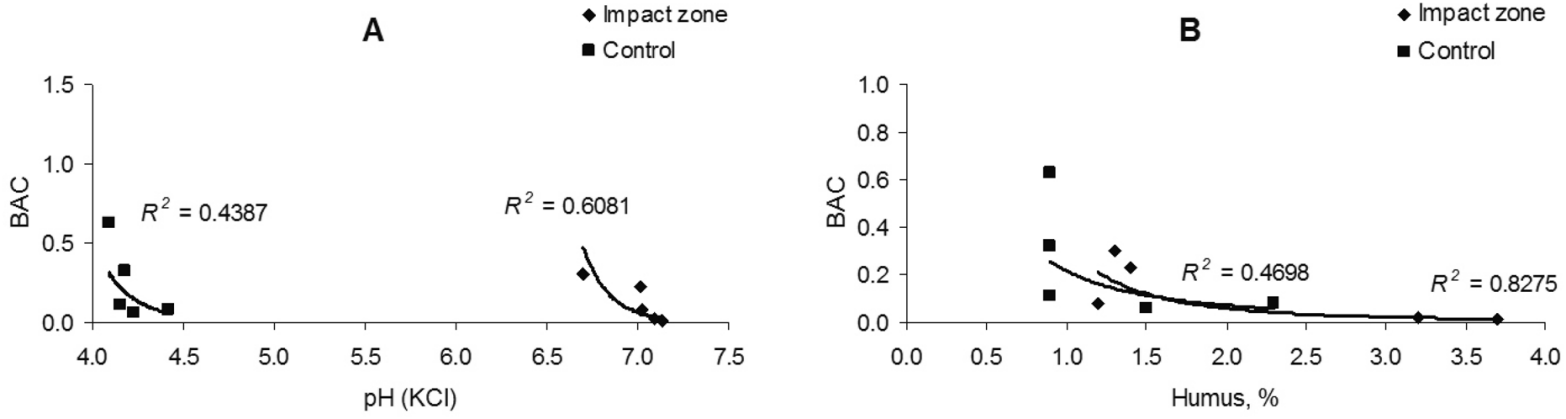
Dependence of the biological accumulation coefficient of manganese on the acidity (**A**) and humus amount (**B**).

The BAC of iron is uncorrelated with soil acidity (*R*^2^ = 0.14–0.18) and the amount of humus (*R*^2^ = 0.05–0.34) (Fig. 9). The BAC of manganese, in contrast, a moderate correspond with the soil acidity (*R*^2^ = 0.43–0.60) and a significant dependent with the amount of humus (*R*^2^ = 0.46–0.82) (Fig. 10).

The ratio of the BAC of iron and manganese (Fe/Mn) in control (except for the surface 0–10 cm soil layer) and in the “relatively clean” 30–50 cm soil layers in the impact zone shows that fine pine roots absorb more manganese than iron (the ratio is 0.12–0.80). However, when the iron and manganese content enlargements in the upper layers of soil, the accumulation pattern shifts towards increased iron absorption (up to 1.1 in control and up to 3.2–6.0 in the impact zone). An utmost difference is observed in the surface 0–10 cm soil layer in the impact zone, where the value of the BAC of iron beats the BAC of manganese by 37.0 times. The ratio of the BMC of iron and manganese (Fe/Mn) for needles and shoot in control is 0.84–1.24, this suggests that iron and manganese from fine roots into shoots and needles migrate equally. But in polluted conditions, this ratio changes and is 0.05–0.07, i.e., the coefficient for iron is 13.04–19.78 times less than for manganese, which indicates that under the contaminated environment the migration pattern shifts towards increased manganese accumulation in the aboveground part of pine trees.

## Discussion

Manganese in plants mainly activates the action of various enzymes (or is part of them), which are of considerable importance in redox processes, photosynthesis, respiration, etc. It catalyzes not only various reactions of carbohydrate cleavage and organic acid metabolism, but also a number of substantial transformations involved in the exchange of nitrogen and phosphorus. When manganese excessive intake into plants, it causes toxic symptoms. Manganese toxicity is one of the primary causes of plant damage when growing on acidic soils^57^. High iron availability in soils can bring about toxicity effects when an excessive uptake of iron damage cell structures, leading to reduced plant growth and injury to foliage^58,59^. High iron toxicity also inhibits nutrient uptake by damaging the epidermis surface of roots^60^.

In the impact zone, the Novlipetsk Steel releases significant contamination of the surface layers of the soil with iron and manganese, while the manganese content in the 0–20 cm soil layer is 27–32 times higher than the background concentrations. Despite the distance from the pollution source, the topmost layers of the soil in the control are also contaminated with iron and manganese. Along the profile of light gray forest soils manganese spreads unevenly, its maximum concentration is observed in the topmost layers, while iron in the identical conditions is distributed more evenly.

There is controversial information about the dependence of iron and manganese content on soil acidity and the amount of humus. So, no correlative relationships between the contents of Mn and humus, Mn and pH in the soils of Dagestan have been found^61^. Contrariwise, it was founded a weak negative correlation exists between the manganese content and soil humus content and pH for soils of some mountain regions^62^. Correlation between the content of mobile iron and some soil factors (pH_КCl_, general and mobile carbon of humus) were revealed for some soil types (e.g., podzolic and sod-podzolic)^63^. We have demonstrated a significant correlation amidst the metal content and soil conditions (acidity and humus amount) just for manganese, but for iron this dependence was a very weakly (uncorrelated) or weak. The iron and manganese contents in fine pine roots are likewise weak or moderate correlated with soil acidity and humus quantity.

According to the value of the BAC and BMC pine does not accumulate iron in fine roots, annual shoots, and needles (both BAC and BMC < 1). However, under conditions of contamination, iron accumulates more intensively in the fine pine roots (the BAC is 1.75–4.22 higher than in the control). Whereas manganese in fine roots under pollution conditions accumulates less than in the control, where the values of the BAC are 2.1–8.0 times higher than in the area of direct influence of emissions of the steel plant (except for the layer of 30–40 cm). Based on the values of the biological accumulation and migration coefficients, we can be marked, that under the contaminated environment the manganese mobility raised, and the translocation pattern shifts towards increased this metal stock in the aboveground part of pine trees.

Analysis of the ratio of iron and manganese absorption confirms the thesis that these metals are antagonists. The antagonistic relationship of these metals may occur either during absorption by roots or during translocation from roots to shoot^64–67^. Thusly, the increased absorption of iron by fine pine roots reduces the supply to the roots of more plant-toxic manganese, but it does not the limit to manganese migration into the aboveground part of the pine plant.

## Conclusion

In the last century, man-made effects and amounts of human-moving chemicals in the biosphere have become comparable to the scale of geological and other natural processes. Counting the number of toxicants entering the environment (including metals) shows that human activity represents currently the significant factor affecting the global and regional cycles of most chemical elements^68^. Metallurgical factories constitute the most extensive sources of toxicants coming into the environment. Their emissions include a significant number of pollutants, including heavy metals. Woody plants, growing in man-made conditions, are forced to adapt to environmental changes.

Studies have shown that the light gray forest soils of the region are excessively contaminated by emissions of the Novlipetsk Steel, primarily manganese (the concentration of which in polluted conditions is 27–32 times higher than the background concentrations). The maximum concentration of metals is detected in the uppermost layers of the soil. The content of iron in the soil and fine pine roots is uncorrelated or weak correlated with the acidity and amount of humus. A significant dependence on soil characteristics is established merely for the manganese content in the soil, while its contents in fine roots are uncorrelated accompanied by soil acidity and the amount of humus (except for contaminated conditions where a moderate correlation with the humus quantity was observed).

Analyzing the values of the biological accumulation and migration coefficients of these metals, it should be mentioned that in the conditions of pollution, there is an increase in the absorption of iron by the fine pine roots, while for manganese, on the contrary, there is a decrease in the values of this coefficient (which indicates less of its absorption by fine roots). These alterations in the ratio of iron and manganese uptake can be explained by their antagonistic relationships.
